# Advances in Induced Pluripotent Stem Cell-Derived Natural Killer Cell Therapy

**DOI:** 10.3390/cells13231976

**Published:** 2024-11-29

**Authors:** Wenhua Qiao, Peng Dong, Hui Chen, Jianmin Zhang

**Affiliations:** 1 CAMS Key Laboratory for T Cell and Immunotherapy, State Key Laboratory of Common Mechanism Research for Major Diseases, Department of Immunology, Institute of Basic Medical Sciences, Chinese Academy of Medical Sciences and School of Basic Medicine, Peking Union Medical College, Beijing 100005, China; 18717598717@163.com; 2 Changzhou Xitaihu Institute for Frontier Technology of Cell Therapy, Changzhou 213000, China; dongpeng204@126.com

**Keywords:** iPSC (induced pluripotent stem cell), cancer immunotherapy, NK cells, gene editing

## Abstract

Natural killer (NK) cells are cytotoxic lymphocytes of the innate immune system capable of killing virus-infected cells and/or cancer cells. The commonly used NK cells for therapeutic applications include primary NK cells and immortalized NK cell lines. However, primary NK cell therapy faces limitations due to its restricted proliferation capacity and challenges in stable storage. Meanwhile, the immortalized NK-92 cell line requires irradiation prior to infusion, which reduces its cytotoxic activity, providing a ready-made alternative and overcoming these bottlenecks. Recent improvements in differentiation protocols for iPSC-derived NK cells have facilitated the clinical production of iPSC-NK cells. Moreover, iPSC-NK cells can be genetically modified to enhance tumor targeting and improve the expansion and persistence of iPSC-NK cells, thereby achieving more robust antitumor efficacy. This paper focuses on the differentiation-protocols efforts of iPSC-derived NK cells and the latest progress in iPSC-NK cell therapy. Additionally, we discuss the current challenges faced by iPSC-NK cells and provide an outlook on future applications and developments.

## 1. Introduction

In recent years, cell therapy has emerged as an advanced and promising treatment modality that has achieved significant success in cancer therapy, particularly for hematological malignancies. The approval of several CAR-T cell products by the FDA underscores the immense potential of cell therapy [1]. T cells are the primary focus of cell therapy research and are considered important immune cells due to their excellent antitumor activity. However, T cells face unresolved limitations, including the potential for cytokine release syndrome (CRS) [2,3,4]; MHC dependency, which may cause graft-versus-host disease (GVHD); and the risk of autoimmune reactions due to cross-pairing with endogenous TCR chains [5,6,7].

NK cells are innate immune cells, due to their strong cytotoxic activity, and can be independent of MHC, and they are considered a safer option for cell-based therapies [8,9,10,11]. In recent years, there has been a surge in research on the antitumor properties of NK cells from various sources and methods to enhance their antitumor functions. Among these, iPSC-derived NK cells offer a promising avenue for NK cell therapy with their broad availability and high uniformity. This paper summarizes the advantages and disadvantages of NK cells from different sources, reviews the current protocols for differentiating iPSC-NK cells and their applications in cancer treatment, and provides an overview of ongoing clinical trials and developed products of iPSC-derived NK cells. We also discuss the challenges this cell therapy faces and its future prospects.

## 2. Application of NK Cells in Tumor Therapy

NK cells originate from common lymphoid progenitors (CLPs), which in turn derive from hematopoietic stem cells (HSCs) in the bone marrow (BM) [8,12,13,14,15,16]. NK cells are found in the bloodstream, comprising 5–15% of circulating lymphocytes, as well as in various lymphoid and non-lymphoid organs, including the spleen, lungs, and liver [1,17,18]. In humans, NK cells are typically defined by the presence of CD56 and the absence of CD3 [19,20,21]. Researchers generated IL-15^−/−^, IL-15Rα^−/−^, and IL-2Rβ^−/−^ mice and observed that deficiencies in IL-15, the IL-15 receptor subunit α (IL-15Rα), or the IL-2 receptor subunit β (IL-2Rβ) resulted in a marked depletion of NK cells in vivo. These findings suggest that IL-15 and IL-2 play crucial roles in NK cell development [22,23,24]. Other studies have shown that IL-2 can promote the development of NK cells in vitro, but it is not necessary for the development of NK cells in mice [25]. The activation receptors governing NK cell cytotoxicity include NKG2D, NKp30, NKp44, and NKp46. The ligands for these activating receptors are proteins such as MHC class I chain-related polypeptide A (MICA) and MICB. MICA and its close relative MICB may signal cellular distress and evoke immune responses [26,27]. NK cells primarily produce Th1-type cytokines [28,29] such as IFN-γ, TNF, and granulocyte-macrophage colony-stimulating factor (GM-CSF) in response to tumor ligands and intracellular pathogens, promoting the activation of immune cells such as dendritic cells (DCs) and T cells. Overall, NK cells play a key role in immune surveillance, and their therapeutic effects against viral infections and tumorigenesis have been demonstrated in both mice and humans [30,31].

Autologous and allogeneic NK cell therapies have shown great potential in cancer treatment. Some early clinical trials have demonstrated that autologous lymphokine-activated NK cells exhibit significant therapeutic efficacy against several types of advanced metastatic cancers [32,33]. In a clinical trial (UMIN000007527), expanded autologous NK cells were shown to safely and effectively treat patients with advanced gastrointestinal cancers [34]. However, some studies have indicated that autologous NK cell therapy does not produce a positive therapeutic effect [35,36], primarily due to a matching of inhibitory receptors on autologous NK cells with the MHC class I presented by cancer cells, subsequently inhibiting NK cell activation [37,38]. Recent studies have suggested that the systemic administration of cytokines can enhance NK cell antitumor activity, overcoming its limitations, with the main strategy in adoptive cancer immunotherapy being systemic cytokine administration combined with NK cells [39,40,41]. Ex vivo IL-2-activated NK cells have shown better results than systemic IL-2 administration [34], but this approach has limited efficacy [42]. Another study demonstrated that in patients with non-Hodgkin lymphoma (NHL), autologous NK cells expanded ex vivo with IL-15 could effectively induce remission [43]. However, high doses of IL-15 can induce cytokine release syndrome (CRS) in patients with advanced acute myeloid leukemia (AML) and dose-limiting toxicity in patients with malignant melanoma or renal cell carcinoma [44,45].

The adaptive transfer of NK cells does not promote graft-versus-host disease (GVHD) as T cells do, significantly enhancing the safety profile of allogeneic NK cell administration. Researchers have demonstrated that alloreactivity between donor and recipient NK cells can eliminate leukemia relapses and graft rejections, while simultaneously protecting patients from GVHD. In mouse models, the pre-transplant infusion of alloreactive NK cells obviated the need for intensive conditioning regimens and reduced the incidence of GVHD [46]. Clinical evidence indicates that allogeneic NK cells demonstrate promising efficacy in controlling high-risk leukemia, renal cell carcinoma, and other human malignancies [10,47,48,49]. A phase I/II clinical trial of anti-CD19 CAR-transduced umbilical cord blood-derived NK cells showed promising results, with an infusion response rate of 73%, no CRS toxicity, and long-term survival [11]. CAR-NK cells are resistant to inhibition mediated by the NKG2A/HLA-E interaction; however, they remain susceptible to inhibition through KIRs, depending on the expression level of HLA class I on the target cells. Even after CAR downregulation, they retain the ability to lyse target cells [50]. Various studies have demonstrated a positive correlation between the number of NK cells in patients’ peripheral blood and prognosis in solid tumors, such as non-small-cell lung cancer (NSCLC), melanoma, and colorectal cancer [51,52,53]. CAR-NK cell therapy can also be applied in the treatment of non-small-cell lung cancer [53]. Combining allogeneic NK cells from healthy donors with the PD-1 inhibitor pembrolizumab can enhance the antitumor immune function of pembrolizumab [54]. NK cell therapy can also be applied to the treatment of liver tumors, with glypican-3 (GPC3) serving as a rational immunotherapeutic target for hepatocellular carcinoma (HCC). Min Yu et al. designed GPC3-targeted CAR-NK-92 cells, which demonstrated effective anti-tumor activity against HCC [55]. Cytokines such as IL-12, IL-15, and IL-18 can enhance the antitumor ability of NK cells in vivo, thereby inhibiting liver tumorigenesis [56]. In a phase I clinical trial evaluating the safety of the intravenous administration of allogeneic NK cells combined with chemotherapy, researchers treated non-small-cell lung cancer (NSCLC) patients with allogeneic NK cells expanded ex vivo using IL-15 and hydrocortisone. The results indicate that repeated doses of up to 2.9 × 10^7^ NK cells/kg per dose and total doses of up to 4.84 × 10^9^ cells were safe, with no signs of GVHD or other local or systemic side effects [57]. Other phase I clinical trials have demonstrated that the ex vivo expansion of NK cells using K562 Clone9.mbIL21 feeder cells to generate allogeneic mbIL21-NK cells for the treatment of leukemia is safe and may effectively control leukemia relapses [58].

Clinical trials with NK cells require the infusion of a large number of cells, ranging from 5 × 10^6^ to 1 × 10^8^ CD3+CD56+ NK cells per kilogram of body weight [59]. This poses significant challenges for the source and expansion of NK cells used for treatment. Another major limitation of NK cells is that their function and activity are easily affected by cryopreservation, limiting their therapeutic feasibility. However, pretreatment with IL-15 and IL-18 can restore the activity of cryopreserved NK cells [60]. Currently, there are four primary sources of NK cells: peripheral blood (PB), umbilical cord blood (UCB) [10,61], human embryonic stem cells (hESCs), induced pluripotent stem cells (iPSCs) [62,63], and NK cell line NK-92 cells [64,65,66]. Most NK cell clinical trials are based on UCB-NK cells, PB-NK cells, and the lymphoma-derived NK cell line NK-92. However, NK cells derived from these sources present certain limitations in terms of manufacturing and storage. While readily available as off-the-shelf products, PB-derived NK cells have high heterogeneity and donor variability and are highly sensitive to cryopreservation. Moreover, the isolation and expansion of PB-derived NK cells can result in mixtures [67]. The NK-92 cell line has been widely used in NK cell therapy due to its high expression of CD56; strong proliferation ability; few inhibitory receptors (NKGA/B, a low level of KIR2DL4, and ILT-2); lack of most killer inhibitory receptors (KIRs) cloned and expressed in normal NK cells; high level of safety; good product homogeneity; and easy gene editing [68,69]. However, NK-92 cells are derived from patients with non-Hodgkin lymphoma [70] and require irradiation before infusion, which greatly reduces their antitumor efficacy post-irradiation. Additionally, NK-92 cells lack CD16 expression and are thus unable to undergo ADCC [68,71]. Moreover, healthy PBMCs, particularly NK cells, have been found to target and lyse NK-92 cells, further reducing the efficacy and survival of these cells in circulation [72]. UCB-derived NK (UCB-NK) cells have high proliferative potential and are available from cord blood banks worldwide. However, UB-NK cells exhibit weak cytotoxicity, immature phenotypes, increased inhibitory NKG2A expression, reduced activating receptor expression, and mixture formation during isolation and expansion [65,66,67].

Contrary to these limitations, iPSC-derived NK cells and NK-92 cells, are homogeneous, because they are derived from clonal populations and are more amenable to engineering [1,70,73]. iPSC-derived NK cells can be generated from sources such as fibroblasts or peripheral blood, making them readily accessible. Furthermore, they maintain homogeneity during the expansion process and can be stored long term [74]. These cells express a range of NK cell surface markers, including a low-frequency subset positive for killer immunoglobulin-like receptors (KIRs); an intermediate-frequency subset positive for killer cell lectin-like receptors (NKG2A, NKG2C, and NKG2D); and CD56 and CD16 [73,75,76,77,78,79,80,81,82]. Therefore, iPSC-derived NK cells can overcome the heterogeneity of other NK cell sources while retaining NK cells’ proliferative and antitumor efficacy and meeting clinical-scale culture requirements. These advantages make iPSC-derived NK cells an ideal candidate for the next generation of NK cell therapies.

## 3. Application of iPSC-NK Cells in Tumor Therapy

The differentiation of iPSCs into NK cells generally follows two stages. The first stage involves inducing iPSCs into CD34+ hematopoietic progenitor cells (HPCs), and the second stage involves the differentiation of these CD34+ HPCs into NK cells. Initially, the Kaufman group discovered that a coculture of human embryonic stem cells (ESCs) with mouse bone marrow stromal cells yielded CD34+CD45+ hematopoietic progenitor cells, which were then sorted and differentiated into NK cells in the presence of SCF, FLT3L, IL-3, IL-15, and IL-7 in a culture medium [77]. Other research groups replaced the second stromal cell line with genetically engineered feeder cells expressing the Notch ligands DLL1 or DLL4, which promote HPC expansion and biased differentiation toward the NK lineage [83,84]. However, culture methods requiring stromal cells for clinical-scale production are costly and require high-quality stromal cell cultivation.

When iPSCs are cultured in a suspension without feeder layers (using low-attachment plates), they spontaneously form aggregates called embryoid bodies (EBs). Cells within EBs respond to exogenous cytokines and differentiate into specific lineages. However, a drawback of EBs is their size variability, which affects their differentiation reproducibility, making it challenging to meet good manufacturing practice (GMP) standards [85]. Knorr et al. developed a “spin EB” method, in which EBs were cultured for 11 days without feeder cells or a serum to obtain CD34+ hematopoietic progenitor cells. These EBs were then transferred to feeder-free plates with an NK differentiation medium containing SCF, FLT3L, IL-3, IL-15, and IL-7, resulting in NK cell differentiation for over 4 weeks [77]. Another group refined the iPSC-to-EB method, reducing the induction time from 11 days to 6 days to obtain CD34+ hematopoietic progenitor cells [86]. These iPSC-derived NK cells from EBs exhibit phenotypes and functions similar to those derived from stromal cells [79]. Importantly, these NK cells demonstrate effective antitumor and antiviral activities both in vitro and in vivo [75].

A common challenge for NK cell therapies, regardless of the source, is their relatively short lifespan, with allogeneic NK cells surviving for only a few weeks. Therefore, various strategies have been developed to enhance NK cell proliferation, in vivo persistence, and tumor-targeting functions. iPSC-derived NK cells have a significant advantage over other NK cell sources due to their amenability to genetic engineering, allowing for the creation of standardized cell populations with unlimited supply once a successful clone is identified. Recent studies have focused on genetically modifying iPSCs, followed by their differentiation into NK cells to obtain engineered iPSC-derived NK cells [87,88,89]. Current strategies to enhance the functionality of iPSC-derived NK cells primarily include the following aspects.

### 3.1. Improving Functional Persistence and Activity

IL-15 promotes NK cell proliferation, differentiation, and activation, and its role in NK cell proliferation has been demonstrated in clinical trials [90,91,92,93,94]. Therefore, increasing IL-15 expression on NK cells is a primary strategy to enhance NK cell proliferation and functional persistence. CIS was the first identified member of the suppressor of the cytokine signaling (SOCS) protein family [95]. CIS, SOCS1, and SOCS3 have been shown to directly bind JAK1 and inhibit JAK–STAT signaling [35,36]. In mice, deletion of Cish enhances IL-4 and IL-2 signaling in CD4+ T cells and increases IL-15 sensitivity in NK cells, improving the control of experimental tumor metastasis [96]. Huang Zhu et al. used CRISPR-Cas9 technology to delete the *CISH* gene in iPSCs, increasing the IL-15-mediated JAK–STAT signaling activity. *CISH*^−/−^ iPSC-NK cells exhibit improved expansion and enhanced cytotoxic activity against various tumor cell lines under low cytokine conditions. In a leukemia xenograft model, *CISH*^−/−^ iPSC-NK cells exhibited significantly increased in vivo persistence and tumor progression [97].

NKG2C is an activating receptor that forms heterodimers with CD94 and signals through the adaptor protein DAP12 [98]. NKG2C+ adaptive NK cells exhibit enhanced functionality against AML, making NKG2C a promising target for NK cell immunotherapy. Jeffery S. Miller’s group created an anti-NKG2C/IL-15/anti-CD33 killing engager (NKG2C–KE) to direct NKG2C+ cells to target CD33+ AML cells. NKG2C–KE induces specific degranulation, interferon-γ production, and the proliferation of NKG2C+ NK cells in patients with reactivated cytomegalovirus after allogeneic transplantation, enhancing the in vivo persistence of adaptive NKG2C+ NK cells. They also tested NKG2C–KE on iPSC-derived NK cells and showed that NKG2C–KE promoted the in vivo persistence of NKG2C+ iPSC-NK cells [99].

Although IL-15 enhances NK cell antitumor functions, high doses of IL-15 may cause toxicity [95,100]. To mitigate this risk, a team led by Jeffrey S. Miller developed a construct named NKG2C–KE, an NKG2C/IL-15/CD33-targeted cytotoxic engager designed to direct NKG2C+ cells toward CD33+ cells, specifically targeting acute myeloid leukemia (AML) cells. These iPSC-derived NK cells exhibited an enhanced serial killing capacity and in vivo persistence without exogenous cytokines, maintaining high levels in peripheral blood for several weeks post-adoptive transfer without additional cytokines [101].

### 3.2. Enhancing NK Cell-Mediated ADCC

NK cell-mediated ADCC is regulated by the Fc receptor FcγRIIIa (CD16a), whose surface expression is modulated by ADAM17 (a disintegrin and metalloprotease-17) [102,103]. Huang Zhu et al. demonstrated that point mutations in CD16a prevent protease-induced surface cleavage. These non-cleavable CD16a variants, including high-affinity non-cleavable CD16a (hnCD16), were engineered into human iPSCs to generate iPSC-derived NK (hnCD16-iPSC-NK) cells. Compared to unmodified iPSC-NK and peripheral blood-derived NK (PB-NK) cells, hnCD16-iPSC-NK cells resist ADAM17-induced CD16a cleavage and exhibit enhanced ADCC against multiple tumor targets [104]. Karrune V. Woan and colleagues generated triple-gene-edited iPSC-NK cells, termed iADAPT cells, engineered to express the high-affinity, non-cleavable Fc receptor CD16a; the membrane-bound IL-15/IL-15R fusion protein; and to knock out CD38, an extracellular enzyme hydrolyzing NAD+. Adaptive NK cells play a dominant role in antibody-dependent cellular cytotoxicity (ADCC) responses, with enhanced ADCC observed following the expansion of adaptive NK cells in individuals with a CMV infection [105,106,107]. iADAPT cells show metabolic and gene expression profiles similar to those of cytomegalovirus-induced adaptive NK cells, persisting in vivo without exogenous cytokines and demonstrating superior antitumor activity [101].

### 3.3. Enhancing Targeting

Enhancing the tumor targeting of iPSC-derived NK cells is primarily achieved by expressing chimeric antigen receptors (CARs). Compared to CAR-T cells, CAR-NK cells have a limited lifespan and a relatively lower risk of on-target/off-tumor toxicity [108]. CAR-NK cell therapy retains the antitumor efficacy of CAR-T cell therapy while mitigating certain risks. Additionally, CAR-NK cells can eliminate cancer cells through CAR-dependent and CAR-independent mechanisms [50]. Given the diverse sources of NK cells, CAR-NK cells can be produced as “off-the-shelf” products, eliminating the need for personalized and patient-specific products that current CAR-T cell therapies require [109]. Numerous preclinical and clinical studies of CAR-NK cells have shown promising therapeutic effects.

iPSC-derived NK cells are easier to genetically engineer than other NK cell sources and can be produced in unlimited, homogeneous quantities for clinical applications. Dan S. Kaufman’s group evaluated various CAR constructs to enhance NK cell-mediated killing and identified a CAR containing the NKG2D transmembrane domain, the 2B4 costimulatory domain, and the CD3ζ signaling domain, which mediates strong antigen-specific NK cell signaling. Compared with PB-NK, iPSC-NK, and T-CAR-iPSC-NK cells, NK-CAR-iPSC-NK cells derived from human iPSCs expressing this CAR exhibit typical NK cell phenotypes and significantly inhibit tumor growth and prolong survival in ovarian cancer xenograft models. These NK-CAR-iPSC-NK cells provide standardized, targeted “off-the-shelf” lymphocytes for anticancer immunotherapy with similar in vivo activity to T-CAR-expressing T cells but with less toxicity [63].

Shin Kaneko’s group used human leukocyte antigen (HLA)-homozygous iPSCs to derive NK/ILC cells expressing a CAR targeting glypican-3 (GPC3) [110]. These cells demonstrated stable CD45/CD7/CAR expression, cytotoxic effector functions, and IFN-γ production, and they showed significant therapeutic effects on gpc3-positive, ovarian tumor, and immunodeficient mice [110]. Melissa Yu et al. engineered iPSC-NK cells expressing the epidermal growth factor receptor (EGFR) CAR, which exhibited antitumor activity in a glioblastoma (GBM) model. In addition to incorporating CARs into iPSC-derived NK cells, many research teams have implemented multiple gene modifications to further enhance the function of iPSC-NK cells [111]. Frank Cichocki and colleagues developed iDuo NK cells, iPSC-derived NK cells engineered to express an anti-CD19 CAR, and a non-cleavable CD16 receptor, along with a membrane-bound IL-15/IL-15R fusion protein to promote in vivo persistence. The iDuo NK cells demonstrated potent CAR-mediated cytotoxicity and, in combination with rituximab, enhanced the targeting of malignant B cells. Compared to anti-CD19 CAR-T cells, iDuo NK cells preferentially target malignant B cells over healthy B cells [112]. Furthermore, Cichocki and colleagues reported a quadruple gene-edited iDuo-MM-CAR-NK cell that incorporates an NK cell-optimized chimeric antigen receptor (CAR) specific for B-cell maturation antigen (BCMA) and a high-affinity, non-cleavable CD16 receptor to enhance antibody-dependent cellular cytotoxicity (ADCC). This design also includes membrane-bound IL-15 to boost functionality and persistence, along with CD38 knockout to prevent antibody-mediated fratricide and improve NK cell metabolic adaptability. iDuo-MM-CAR-NK cells demonstrated tumor control comparable to primary anti-BCMA CAR-T cells both in vitro and in vivo without complications, such as graft-versus-host disease (GVHD). Notably, when combined with daratumumab (an anti-CD38 monoclonal antibody), iDuo-MM-CAR-NK cells achieved maximal ADCC activity and anti-tumor efficacy against multiple myeloma (MM) [113]. Xiaolan Lin et al. developed iPSC-NK cells expressing a CAR targeting MUC1, which demonstrated therapeutic efficacy against human oral tongue squamous cell carcinoma (OTSCC) both in vitro and in xenograft models in BNDG mice. This suggests that CAR-iNK cells represent a potential therapeutic tool for solid tumors [114].

### 3.4. Inhibition of Immune Checkpoints

CD155/T-cell immunoreceptor with Ig and ITIM domains (TIGIT) is a novel immune checkpoint. CD155, an adhesion molecule upregulated during tumor progression, promotes tumor cell proliferation and migration through various pathways. TIGIT is an inhibitory receptor primarily expressed on natural killer (NK) cells, CD8+ T cells, CD4+ T cells, and regulatory T (Treg) cells. CD155 interacts with the inhibitory checkpoint receptor TIGIT to transmit immune signals, thereby inhibiting the function of T cells and NK cells [115]. Kyle B. Lupo et al. engineered a synNotch-mediated activation of iPSC-NK cells to inhibit the TIGIT–CD155 axis, facilitating the activation of downstream signaling cascades mediated by transcription factors, leading to a controlled local blockade of CD73. This “decoy” receptor binds CD155 to TIGIT but skews the inhibitory TIGIT/CD155 interaction toward activation via downstream synNotch signaling. The disruption of TIGIT and CD73 activities promoted the function of iPSC-NK cells after adoptive transfer into intracranial patient-derived glioblastoma models, enhancing their innate cytolytic function against the tumor and ultimately eradicating the tumor [116].

## 4. Clinical Studies of iPSC-NK Cells

Numerous global clinical studies are focusing on genetically engineered iPSC-derived NK cells. Notably, FT576 and FT522 (both from Fate Therapeutics) and CNTY-101 (Century Therapeutics) have entered the clinical stage. FT576 is an investigational off-the-shelf CAR NK cell cancer immunotherapy derived from iPSC lines. FT576 incorporates four functional modifications: a proprietary CAR targeting BCMA; a novel high-affinity, non-cleavable CD16 (hnCD16) Fc receptor, engineered to enhance ADCC activity; an IL-15 receptor fusion protein (IL-15RF) to promote NK cell activity; and the elimination of CD38 expression to reduce the potential for NK cell fratricide [117]. CD38 is highly expressed on the surface of multiple myeloma cells, and daratumumab, which targets the surface protein CD38 on these cells, has shown single-drug efficacy in relapsed/refractory myeloma. Daratumumab can induce the death of myeloma cells through antibody-dependent cellular cytotoxicity (ADCC), but because CD38 is also expressed on the surface of NK cells, daratumumab treatment can induce the self-destruction of NK cells that mistakenly target CD38-expressing NK cells, which may weaken the effectiveness of ADCC-mediated targeting and the elimination of myeloma [118]. An ongoing phase I clinical trial (NCT05182073) is evaluating FT576 cells as a monotherapy or in combination with daratumumab for the treatment of patients with relapsed/refractory multiple myeloma. Preliminary phase I results indicate that after two treatment cycles, four patients achieved stable disease (SD), two patients showed progressive disease (PD), one patient experienced a very good partial response (VGPR), one patient demonstrated minimal response (MR), and one patient achieved partial response (PR) [119].

FT522 cells integrate five novel synthetic controls of cellular functionality, designed to enable the dual antigen targeting of CD19 and CD20 antigens expressed on B cells: hnCD16 to enhance ADCC activity; IL-15RF for NK cell persistence, without the need for exogenous cytokine support; the elimination of CD38 expression to reduce the potential for NK cell fratricide; and an incorporation of the company’s alloimmune defense receptor (ADR) technology to promote functional persistence [120,121].

CNTY-101 employs Allo-Evasion™ technology to delete β2M, eliminating HLA-II expression to prevent autologous T-cell killing; includes HLA-E expression to bind NKG2A, preventing killing by autologous NK cells; incorporates the homeostatic cytokine IL-15 to enhance persistence and functionality; and features an EGFR safety switch that allows for the targeted elimination of EGFR-expressing cells through ADCC via a cetuximab injection, which is designed to facilitate the rapid clearance of infused cells, if necessary [122]. CNTY-101 is currently being evaluated in the phase I ELiPSE-1 trial (NCT05336409) to treat relapsed or refractory CD19-positive B-cell lymphoma. Additionally, a phase I CALiPSO-1 trial (NCT06255028) will be conducted for the treatment of moderate-to-severe systemic lupus erythematosus [123]. Table 1 summarizes the ongoing clinical trials.

## 5. Summary and Outlook

With the continuous development of CAR-T cell therapy, CAR-NK cell therapy has also attracted attention due to its high safety. iPSC-NK cells are expected to become the mainstream of NK cell therapy products in the future because of their easy access, easy genetic modification, and the ability to maintain product homogeneity. In recent years, various CAR-iPSC-NK cell products obtained by engineering iPSC-NK cells have shown encouraging therapeutic effects on hematological and solid tumors in preclinical and clinical trials. Although iPSC-NK cells exhibit significant potential and advantages in cancer therapy, several issues must be addressed before they can be clinically applied.

First, a fundamental concern for all iPSC-derived cells is safety. The risk of tumorigenesis associated with iPSCs and the stability of iPSCs during culturing are critical issues that need to be resolved [124,125]. Second, although the “spin EB” culture method has optimized the induction and differentiation process of iPSC-NK cells, achieving a clinical-scale production of iPSC-NK cell products requires standardizing the entire process of induction, differentiation, activation, and expansion to ensure product homogeneity. Additionally, genetic modifications using technologies such as CRISPR/Cas9 to produce CAR iPSC-derived NK cells have raised concerns about product safety [126,127]. Cost is another significant factor to consider. The induction and differentiation process involves the addition of multiple cytokines, and the GMP-grade materials required for clinical-scale production significantly increase costs. Finally, while iPSC-NK cell therapies have shown promising results in treating hematologic malignancies and lymphomas, their efficacy in treating solid tumors remains suboptimal. Therefore, exploring iPSC-NK cell therapies for solid tumors presents a further challenge.

Despite these challenges, iPSC-NK cells offer substantial potential for the next generation of cellular therapy products, advancing the field of cancer treatment.

## Figures and Tables

**Table 1:** Ongoing clinical trials of iPSC-derived NK Cells.

NCT Number	Product	Stage	Conditions
NCT06027853	Natural killer (NK) cell therapy targeting CLL1 in acute myeloid leukemia	Recruiting	AML; adult
NCT05182073	FT576 (IL-15RF/*CD38*KO/anti-BCMA CAR) iPSC-derived NK cells with daratumumab	Recruiting	Multiple myeloma; myeloma
NCT03841110	Nontransduced iPSC-derived NK cells (FT500) with checkpoint blockade	Completed	Advanced solid tumors; lymphoma; gastric cancer; 15 more
NCT04363346	FT516 (hnCD16) iPSC-derived NK cells	Completed	COVID-19
NCT05700630	Ph1 study of FT538 alone and with vorinostat for persistent low-level HIV viremia	Not yet recruiting	HIV-1 infection; ART; Cd4+ lymphocyte deficiency; 2 more
NCT04630769	FT516 (hnCD16) iPSC-derived NK cells with enoblituzumab and IL-2	Completed	Ovarian cancer; fallopian tube adenocarcinoma; primary peritoneal cavity cancer
NCT05336409	A study of CNTY-101 in participants with CD19-positive B-cell malignancies (ELiPSE-1)	Recruiting	R/R CD19-positive B-cell malignancies; indolent non-Hodgkin lymphoma; aggressive non-Hodgkin lymphoma
NCT04714372	FT538 (hnCD16/*CD38*KO/IL-15RF) iPSC-derived NK cells with daratumumab	Active; not recruiting	AML
NCT04555811	FT596 (hnCD16/anti-CD19 CAR/IL-15RF) iPSC-derived NK cells with rituximab	Active; not recruiting	NHL; diffuse large B-cell lymphoma; high-grade B-cell lymphoma

## Data Availability

These data are available at Pubmed and clinical trails.
